# IDE Gene Polymorphism Influences on BPSD in Mild Dementia of Alzheimer's Type

**DOI:** 10.1155/2008/858759

**Published:** 2008-12-04

**Authors:** Noriko Sato, Akinori Ueki, Hideo Ueno, Hidetaka Shinjo, Yoshio Morita

**Affiliations:** ^1^Department of Neuropsychiatry, Hyogo College of Medicine, Hyogo 663-8501, Japan; ^2^Kaede Cocorono Hospital, Osaka 598-0002, Japan

## Abstract

Insulin degrading enzyme (IDE) degrades amyloid *β* (A*β*), which may inhibit the accumulation of A*β* in a brain affected with dementia of Alzheimer's type (DAT). A decrease in the activity of IDE results in changes in glucose utilization in the brain, which could affect the cognitive and psychiatric symptoms of DAT. We investigated a possible association of IDE gene polymorphism and the behavioral and psychological symptoms of dementia (BPSD) in mild DAT. The genotyping for IDE and apolipoprotein E (ApoE) was determined in 207 patients with mild DAT and 215 controls. The occurrence of BPSD was demonstrated using the Behavioral Pathology in Alzheimer's Disease Rating Scale (BEHAVE-AD). IDE gene polymorphism is unlikely to play a substantial role in conferring susceptibility to DAT, but it may be involved in the development of affective disturbance through the course of mild DAT, regardless of the presence of an ApoE *ε*4 allele. The present data could be the result of a small sample size. Further investigations using larger samples are thus required to clarify the correlation between IDE gene polymorphism, susceptibility to DAT, and emergence of BPSD.

## 1. Introduction

Dementia of Alzheimer's type (DAT) is a progressive degenerative disorder in which the behavioral and psychological symptoms of dementia (BPSD) are unavoidable. These symptoms constitute a great burden not only on the patients, but also on their families and caregivers [1]. The stress levels of caregivers may be reduced by predicting the occurrence of DAT and the development of DAT-associated BPSD.

The characteristic causes of neurological pathogenesis in DAT are amyloid plaque deposition and neurofibrillary abnormalities in the brain. Amyloid plaque is made up of amyloid *β* (A*β*) proteins, which are formed by the proteolysis of amyloid precursor protein. Excessive production and insufficient clearance of A*β* lead to its extracellular deposition as plaque. One way in which A*β* metabolism can fail is through hyperinsulinemia, caused by the increase of insulin resistance through aging, because insulin stimulates A*β* secretion to the extracellular space. One enzyme that regulates A*β* is insulin degrading enzyme (IDE) [2–5].

IDE is concentrated in the cytoplasm—and even more so in the peroxisomes in liver, kidney, muscle, and brain cells—and it plays a key role in degrading many important peptides [6–8]. Therefore, not only DAT but also type 2 diabetes mellitus (DM) are linked to IDE [9, 10], and DM is reported to promote the incidence of DAT [11].

Other studies have already shown that IDE is linked to DAT. Neurological investigations have found that the expression of IDE is reduced in the brains of patients with DAT [12]. Several genetic linkage studies have discovered the existence of a susceptibility locus for Alzheimer's disease on chromosome 10 [13–15]. The IDE gene, located near the distal end of linkage region (10q23–q25), is an attractive candidate for involvement in these phenotypes [16–19]. However, conflicting negative results have been reported [20–26].

In our study, we analyzed SNPrs1999764, which is located in the intron 1 of the IDE gene and which occurs as the consequence of a mutation from thymine (T) to cytosine (C). We used this SNP because it is more frequently polymorphic in the Japanese population than other SNPs [23]. This SNP has not been shown to result in a clear functional change, but intronic SNP generally caused a change in the expression of the protein and it affects the quantity and function of the gene. Therefore, the possible roles of this SNP may lead to a reduction in IDE activity and could be regarded as genetic determinants of predisposition and the development of DAT-associated psychopathology.

We investigated the relationship between IDE gene polymorphism and the occurrence of DAT, the development of DAT-associated BPSD, and BPSD in patients with sporadic mild DAT, independent of apolipoprotein E (ApoE) *ε*4 status.

An improved understanding of the relationship between DAT and IDE gene polymorphism (including the presence of the ApoE *ε*4 allele which is considered to be a risk factor for sporadic DAT) could allow us to predict the occurrence of DAT and the incidence of BPSD by detecting individual polymorphisms in the gene for IDE. This would reduce the burdens on patients, their families, and their caregivers, and would also help to provide more appropriate care to patients in the future.

## 2. Materials and Methods

### 2.1. Subjects

**Figure 1:**
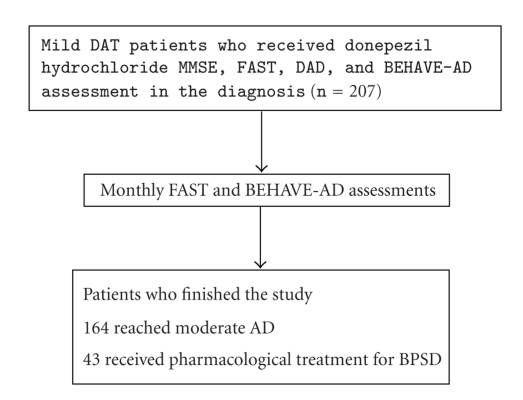
Study flow chart. MMSE: Mini-Mental State Examination; FAST: Functional Assessment Staging; DAD: Disability Assessment for Dementia; BEHAVE-AD: Behavioral Pathology in Alzheimer's Disease Rating Scale.

All patients were collected prospectively from outpatients of the Department of Neuropsychiatry, Hyogo College of Medicine. Two hundred and seven subjects (73.5 ± 8.2 years, 64 male/143 female) with mild DAT met the NINCDS-ADRDA criteria for probable Alzheimer's disease and were assigned to stage 4 on the functional assessment staging (FAST) scale [27]. Each patient received a cognitive assessment using the mini-mental state examination (MMSE) at the time of DAT diagnosis. Functional assessments included the disability assessment for dementia (DAD), which evaluates instrumental and basic activities of daily living [28]. Mean (±standard deviation) duration of follow-up was 2.2 ± 0.7 years until DAT reached a moderate degree, defined as FAST stage 5. According to the FAST scale, this duration of follow-up was consistent with the typical duration of mild dementia in the clinical progression of DAT [27]. All patients were receiving donepezil hydrochloride. During the mild stage of DAT, prevalence of BPSD was determined in semistructured interviews with the subjects' caregivers using the Behavioral Pathology in Alzheimer's Disease Rating Scale (BEHAVE-AD) at the time of DAT diagnosis, then checked once a month to obtain information about only the presence, not the magnitude, of paranoid and delusional ideation, hallucinations, activity disturbance, aggressiveness, diurnal rhythm disturbance, affective disturbance, and anxieties and phobias. We excluded patients with any history of psychiatric hospitalization or use of medication for psychiatric problems at any time before the first evaluation, as well as patients with other forms of dementia such as dementia with Lewy bodies. The prevalence of BPSD was 90 DAT patients (43.5%) at time of diagnosis, but all patients were free of pharmacological treatment for BPSD. Forty-three patients who were treated with pharmacological agents for the management of BPSD during follow-up were excluded from the study. However, the data taken from these patients before their pharmacological treatment began were enrolled in the study. The flow chart of the progress through the study is depicted in Figure 1.

We also collected 215 control subjects (72.1  ± 8.6 years; *t* = 1.672, *P* = .0953, 85 male/130 female; *χ* ^2^ = 3.428, *P* = .0641) whose age and gender were matched. They were interviewed to exclude individuals with a history or evidence of abuse, neurological disease or psychiatric disorders and recruited from the general community or from among medical and care staff volunteers in hospitals or health service facilities. The presence of normal general cognitive function in these subjects was identified by a score of greater than 27 on the MMSE.

These interviews and diagnoses were performed by experienced psychiatrists who were experts for elderly patients with cognitive disorders. The patients were followed by the same psychiatrists.

This study was approved by the ethics committee of the Hyogo College of Medicine. We gave all subjects, who were native speakers of Japanese, a sufficient explanation for the purpose of the study and obtained their voluntary and written informed consent. In the case of DAT, we also gave this explanation to the legal guardian.

### 2.2. Genotyping Method

We took blood from all subjects, injected it into EDTA-containing tubes, and extracted genomic DNA from those samples using an Easy DNA kit (TALENT, Trieste, Italy). We examined IDE gene polymorphism using a polymerase chain reaction restriction fragment length polymorphism (PCR-RFLP) method. In other words, we amplified the target DNA fragment by PCR, using the primers reported by Boussaha et al. [21], and digested it with Alw26I (Fermentas Life Sciences, Ontario, Canada) restriction enzyme. The DNA sequences possessing a T allele were not recognized by Alw26I; those possessing a C allele were recognized. We also analyzed ApoE polymorphism, which has been found to be a serious risk factor for a sporadic DAT onset [29].

### 2.3. Statistics

We evaluated the results among the groups by *χ* ^2^ test for enumerated data with no ordered relationship, while Student's *t*-test was used for averaged data. For all comparisons, values of *P* < .05 were considered statistically significant. In comparisons between polymorphisms and the evaluation of each symptom according to BEHAVE-AD, when we obtained significant results by *χ* ^2^ testing, we required a stricter statistical significance in accordance with the Bonferroni correction.

## 3. Results

### 3.1. Frequency of IDE Gene Polymorphism and Allele

The PCR-RFLP analysis showed that both the DAT and the controls possessed the T allele more frequently than the C allele (genotype distribution: *χ* ^2^ = 3.006, *P* = .2225, allele frequency: *χ* ^2^ = 6.167 × 10^−5^, *P* = .9937). When we ruled out the DAT patients and controls who carried an ApoE *ε*4 allele, we got a similar result (genotype distribution: *χ* ^2^ = 5.397, *P* = .0673, allele frequency: *χ* ^2^ = 0.2991, *P* = .5845). We concluded that the incidence of DAT had no relevance to IDE gene polymorphism and allele frequency, regardless of ApoE *ε*4 status (see Table 1).

### 3.2. Expression Frequency of BPSD

In the course of FAST stage 4, 145 DAT patients experienced some BPSD (70.0%). The most frequently observed symptoms were affective disturbance (32.9%), followed by anxieties and phobias (26.6%), aggressiveness (24.2%), paranoid and delusional ideation (20.8%), activity disturbance (15.0%), hallucinations (12.6%), and diurnal rhythm disturbance (10.1%). This observation may differ from the results obtained by Reisberg et al. [30]. The result indicated that we gave a referral center consultation service that received many requests to see subjects with BPSD.

### 3.3. BPSD Expression and IDE Gene Polymorphism

Among the patients with BPSD, we compared BPSD expression and IDE gene polymorphism, but we could not get significance (genotype distribution: *χ* ^2^ = 3.144, *P* = .2077, allele frequency: *χ* ^2^ = 3.363, *P* = .0667). We then excluded the patients who possessed an ApoE *ε*4 allele, but we still could not get significance (genotype distribution: *χ* ^2^ = 4.056, *P* = .1316, allele frequency: *χ* ^2^ = 3.363, *P* = .0667). The presence of an ApoE *ε*4 allele has been assumed to be involved in the clinical expression of DAT, including BPSD. Scarmeas et al. [31] have reported a possible association between the ApoE *ε*4 allele and BPSD in DAT patients. We found that IDE gene polymorphism does not correlate with the expression of BPSD in patients with or without ApoE *ε*4.

### 3.4. Analysis of BEHAVE-AD Items and IDE Gene Polymorphism

We tabulated the analysis of BEHAVE-AD items and IDE gene polymorphism, which was evaluated during the course of mild stage of DAT (see Table 2). We analyzed the associations between IDE gene polymorphism and the presence of BPSD without the consideration of the magnitude of symptoms. We did not investigate the difference in severity of a wide variety of BPSD according to IDE gene polymorphism. After applying the Bonferroni correction, we obtained a statistically significant result pertaining to the affective disturbance item. The DAT patients who experienced some affective disturbance tended to have T/C and C/C gene polymorphism and an increased incidence of the C allele. Except for those carrying the ApoE *ε*4 allele, the number of subjects who exhibited T/C or C/C gene polymorphism or who possessed the C allele was greater among those patients with affective disturbance. The tendency toward affective disturbance among DAT patients possessing the C allele of the IDE gene was independent of their ApoE *ε*4 status (see Table 3). We also compared the T/T gene polymorphism and C allele among the items of BEHAVE-AD, only affective disturbance was significant with or without ApoE *ε*4 (*χ* ^2^ = 14.07, *P* < .001; *χ* ^2^ = 15.75, *P* < .001).

### 3.5. Background and Preparation Factors of Affective Disturbance Expression

We set out to determine whether any demographic, disease-related, or caregiver-related variables could be affecting the incidence of affective disturbance. We categorized the DAT patients by presence or absence of affective disturbance, and we compared several variables across these groups, beginning with gender (*χ* ^2^ = 2.588, *P* = .1077), education (*t* = 0.7520, *P* = .4530), and cognitive function by means of the MMSE (*t* = 1.499, *P* = .1354). Because social and physical factors such as patient's environment and caregiver contribute to the expression of BPSD, we also compared assessment of ADL (*t* = 1.627, *P* = .1053), incidence of physical complications (*χ* ^2^ = 2.039, *P* = .1533) such as diabetes mellitus (*χ* ^2^ = 0.0990, *P* = .7527) and hypertension (*χ* ^2^ = 0.1040, *P* = .7471), receipt of social welfare services (*χ* ^2^ = 0.0980, *P* = .7547), age of the patient's primary caregiver (*t* = 1.452, *P* = .1479), and the primary caregiver's relationship to the patient (*χ* ^2^ = 2.458, *P* = .4829). But in none of these cases was there a statistically significant difference between DAT patients with affective disturbance and DAT patients without (see Table 4).

## 4. Discussion

Although we hypothesized that there was a correlation between IDE gene polymorphism and the susceptibility to DAT, our study has shown that there is none. There is a correlation, however, between IDE gene polymorphism and one symptom frequently associated with DAT.

We began by examining earlier studies about any substantial roles of IDE gene polymorphism in conferring susceptibility to DAT. One research group has insisted that there was a correlation between increased risk of DAT and IDE gene polymorphism in DAT patients without the ApoE *ε*4 allele [16, 22]. Another report showed that DAT patients with the particular IDE gene polymorphism and ApoE *ε*4 allele developed DAT [17], and others demonstrated a relationship between increased risk of DAT and IDE gene polymorphism that was independent of ApoE *ε*4 allele status [18, 19]. Other reports, to the contrary, insisted that a particular SNP of the IDE gene was not pertinent to the risk of DAT [20, 21, 23–26] (see Table 5). Our study could not identify any relationship. The disagreement among the conclusions of all these authors is probably due to the fact that different authors have examined different SNPs, as well as the fact that the frequency of gene polymorphism and the distribution of alleles vary among regions and races. In fact, compared with the SNP in our study, the Japanese population has a greater frequency of the C allele in comparison with other races (NCBI Single Nucleotide Polymorphism Cluster Report: rs 1999764).

After reviewing the literature, we hypothesized that BPSD expression was affected by IDE gene polymorphism. Because BPSD is used as a measure of the severity of dementia but is assessed only at particular times during the evaluation of DAT, the genetic predisposition could be missed if BPSD was not being expressed at the time of the patient's assessment and assignment to a particular stage of DAT. Thus a longitudinal study of multiple patients with the same degree of DAT was necessary.

There is a significant discrepancy between the estimations of prevalence of BPSD among patients with DAT in different stages [32]. Other studies have shown that the frequency of BPSD may reach a peak during the mild or moderate of the illness and may gradually decrease as the patient deteriorates intellectually and physically [33–35]. Therefore, we assessed BPSD expression among DAT patients at same stage. Our association study of BPSD used repeated measurements across the course of mild DAT. We chose to study patients diagnosed with mild DAT because it is comparatively easy to follow up with individual patients through the whole period of mild dementia and to collect a large number of patients for a longitudinal study. Our results showed that IDE gene polymorphism had a significant relevance to BPSD, specifically to affective disturbance. Nevertheless, no researcher has investigated this correlation before us; therefore, we could not compare our results with others'.

We hypothesized that IDE would have high affinity with insulin and that its concentration could be regulated by glucose metabolism. An earlier study found that glucose metabolism in one part of the cerebral cortex decreased in DAT patients with BPSD, especially those experiencing affective disturbance [36]. In addition, glucose utilization decreases with aging [37]. Moreover, there is a significant interaction between diabetes and depression [38], as well as between diabetes and neurotransmitters such as serotonin [39]. This is why only affective disturbance showed a significant link to IDE gene polymorphism. Thus we can conclude that glucose metabolism and the actions of some neurotransmitters can affect BPSD expression.

In this study, we tried to make a comparison of some background and preparation factors such as physical complications, living environment, and caregiver relationship between the DAT patients with affective disturbance and those without. However, we could not obtain any clear results. Although we hypothesized that glucose metabolism was associated with BPSD expression, the incidence of diabetes mellitus did not correlation with the incidence of BPSD. Perhaps this is because blood glucose, which is a primary energy source for our brain, can be regulated by the hypothalamic-pituitary-adrenal compensatory system even if glucose metabolism becomes imbalanced [40]. As stated above, we could not obtain any significant result, but if a regulated system gradually becomes unwell with aging, the prevalence of diabetes mellitus can rise.

Genetic, neurobiological, psychological, and social factors can affect the expression of BPSD, but in our study, we have concluded that the genetic and neurobiological factors have the greatest influence. For example, the possession of the C allele of the IDE gene can cause patients to develop BPSD such as affective disturbance. This result may prove that this SNP is in linkage disequilibrium and works as a functional alteration of IDE, disrupting the balance of glucose metabolism and neurotransmitter action. These changes can lead to BPSD expression.

The present data could be the result of small sample size, small effect size of the polymorphisms, and the resultant limitation in statistical power. The need for replication using larger samples might be highlighted. However, previous studies investigating IDE polymorphisms for the incidence of DAT have used subject population of 80 and 1217. Moreover, positive results in 80 and 210 DAT patients have been published. Thus the sample size in the present study is not unusually small (see Table 5), and our findings should not be declared spurious on that account.

Throughout this study, we found it difficult to continue examining the same patients due to their age and medical condition, and we might not have been able to finish the study without the cooperation of patients' families. However, being able to predict the expression of certain peripheral symptoms will enable caregivers to prepare the necessary care systems and services, and will enable patients to make the necessary adjustments to their living situations. Further investigations into the correlation between IDE gene polymorphism, susceptibility to DAT, and emergence of BPSD will help even more, so we intend to continue these studies in the future.

## 5. Conclusions

IDE gene polymorphism is unlikely to play a substantial role in conferring susceptibility to DAT, but it may be involved in the development of affective disturbance through the course of mild DAT, regardless of the presence of an ApoE *ε*4 allele.

## Figures and Tables

**Table 1:** Genotype distributions and allele frequencies of IDE gene polymorphism in DAT patients and controls after stratification according to ApoE *ε*4 allele.

	IDE		IDE without ApoE *ε*4	
	DAT	Controls	DAT	Controls
Gene polymorphism				
* *T/T (%)	136 (65.7)	136 (63.2)	69 (67.0)	113 (65.7)
* *T/C (%)	62 (30.0)	75 (34.9)	27 (26.2)	56 (32.6)
* *C/C (%)	9 (4.3)	4 (1.9)	7 (6.8)	3 (1.7)
* * *χ* ^2^ value	3.006		5.397	
* * *P*-value	.2225		.0673	

Allele frequency				
* *T allele, %	80.7	80.7	80.1	82.0
* *C allele, %	19.3	19.3	19.9	18.0
* * *χ* ^2^ value	6.167 × 10^−5^		0.2991	
* * *P*-value	.9937		.5845	

**Table 2:** *χ* ^2^ analysis between IDE genotype and BEHAVE-AD items.

BEHAVE-AD items	IDE	IDE without ApoE *ε*4
	*χ* ^2^ * P*-value	*χ* ^2^ * P*-value
Paranoid and delusional ideation	0.5681	0.6156
Hallucinations	0.5048	0.9484
Activity disturbance	0.3179	0.4907
Aggressiveness	0.1306	0.3513
Diurnal rhythm disturbance	0.0618	0.0770
Affective disturbance	0.0018*	0.0007**
Anxieties and phobias	0.3837	0.2044

*P = .0126; **P = .0049 after Bonferroni correction.

**Table 3:** Genotype distributions and allele frequencies of IDE gene polymorphism in DAT patients with and without affective disturbance after stratification according to ApoE *ε*4 allele.

	IDE			IDE without ApoE *ε*4		
	DAT with affective disturbance		DAT without affective disturbance	DAT with affective disturbance		DAT without affective disturbance
Gene polymorphism						
* *T/T (%)	33 (48.5)		102 (73.4)	15 (41.7)		53 (79.1)
* *T/C (%)	30 (44.1)		33 (23.7)	17 (47.2)		11 (16.4)
* *C/C (%)	5 (7.4)		4 (2.9)	4 (11.1)		3 (4.5)
* * *χ* ^2^ value		12.66			14.66	
* * *P*-value		.0018*			.0007**	

Allele frequency						
* *T allele, %	70.6		85.3	65.3		87.3
* *C allele, %	29.4		14.7	34.7		12.7
* * *χ* ^2^ value		12.48			14.01	
* * *P*-value		.0004^+^			.0002^++^	

*P = .0126; **P = .0049, *^+^P* = .0028; *^++^P* = .0014 after Bonferroni correction.

**Table 4:** Comparison of demographic, disease-related, and caregiver-related variables of DAT patients with and without affective disturbance at the time of diagnosis.

Patients	DAT with affective disturbance	DAT without affective disturbance
Gender, male/female	16/52	48/91
Education, years	11.8 ± 8.1	11.0 ± 5.8
Mean MMSE score ±SD	19.7 ± 3.2	19.0 ± 3.5
Mean DAD score ±SD	80.0 ± 6.9	77.9 ± 9.6
Physical problems, %	29.4	39.6
Diabetes mellitus, %	7.4	8.6
Hypertension, %	16.2	18.0
Receipt of social welfare services, %	32.4	30.2

Mean caregiver's age ±SD, years	56.8 ± 14.6	60.2 ± 14.9
Relationship, % (spouse/child/daughter-in-law/sibling)	(47.1/33.8/16.2/2.9)	(56.8/30.9/10.1/2.2)

**Table 5:** Published studies of association between DAT onset and IDE gene polymorphism.

Author (year)	No. of DAT	No. of control	Association between the risk of DAT and IDE gene polymorphism	SNP (NCBI dbs.rs.no.)
Abraham et al. [20]	86	94	No association	3758505, 464953, 4646954, 4646958
Boussaha et al. [21]	202	186	No association	1999764, 1855916
Edland et al. [16]; Edland [22]	80	118	Association among DAT patients without ApoE *ε*4	3758505, 4646954, 4646958
Sakai et al. [23]	240	163	No association	1999764, 551266
Bian et al. [17]	210	200	Association among DAT patients with ApoE *ε*4	4646953
Nowotny et al. [18]	1217	1257	Association among DAT patients regardless of ApoE *ε*4	2251101
Cellini et al. [24]	302	164	No association	3758505, 4646958
Ozturk et al. [25]	1012	771	No association	2251101, 551266, 1832196
Marlowe et al. [26]	179	516	No association	3758505, 4646954, 1832196, 4646958, 1544210
Mueller et al. [19]	444	269	Association among DAT patients	11187007, 2149632, 7084090, 11187033, 11187033, 11187040, 11187060, 12412249, 7076966
